# Direct and Indirect Influence of Altruistic Behavior in a Social Network

**DOI:** 10.1371/journal.pone.0140357

**Published:** 2015-10-15

**Authors:** Pei-Pei Liu, Vasiliy Safin, Barry Yang, Christian C. Luhmann

**Affiliations:** Department of Psychology, Stony Brook University, Stony Brook, New York, United States of America; Middlesex University London, UNITED KINGDOM

## Abstract

Prior research has suggested that recipients of generosity behave more generously themselves (a direct social influence). In contrast, there is conflicting evidence about the existence of indirect influence (i.e., whether interacting with a recipient of generosity causes one to behave more generously), casting doubt on the possibility that altruistic behavior can cascade through social networks. The current study investigated how far selfish and generous behavior can be transmitted through social networks and the cognitive mechanisms that underlie such transmission. Participants played a sequence of public goods games comprising a chain network. This network is advantageous because it permits only a single, unambiguous path of influence. Furthermore, we experimentally manipulated the behavior of the first link in the chain to be either generous or selfish. Results revealed the presence of direct social influence, but no evidence for indirect influence. Results also showed that selfish behavior exerted a substantially greater influence than generous behavior. Finally, expectations about future partners’ behavior strongly mediated the observed social influence, suggesting an adaptive basis for such influence.

## Introduction

Social dilemmas are pervasive, forcing individuals to choose between maximizing personal benefits to the detriment of others and sacrificing personal welfare for the benefit of others. Altruism, costly behaviors that provide benefits to others, is surprisingly common. This observation has led researchers to rethink assumptions about purely self-interested agents [1, 2] and spurred an extraordinary effort to further examine these apparently altruistic behaviors (for reviews, see [3, 4]). Much of this work has sought to understand the mechanisms that underlie altruistic behavior in hopes that such an understanding will yield strategies to promote altruistic behavior in otherwise selfish individuals.

A great deal of work has explored the idea that social structures can influence a wide variety of behaviors, including behavior in games of cooperation [5–9], dictator games [10–13], trust games [14], coordination problems [15], and exchange markets [16]. With regards to social dilemmas, there is growing evidence that behavior in games of cooperation is influenced by social structures [5–9]. For example, unconditionally altruistic individuals can survive if they exclusively interact with a fixed set of partners [5, 6, 17]. A recent study demonstrated that a relatively small amount of social structure allows conditional altruism to dominate a wide variety of behavioral strategies, including selfish behaviors that seek to exploit altruistic agents [18]. Social stability is not the only circumstance in which altruism is possible. Rand and colleagues have recently explored dynamic social networks: contexts in which individuals have substantial influence over who they interact with [19, 20]. Their findings suggested that such circumstances encourage altruistic behavior because agents are allowed to establish and maintain interactions with cooperative partners while terminating interactions with selfish partners.

Even in the absence of network structure, social factors have been found to exert strong influences on a variety of behaviors [21–24]. The idea of social influence has a long history in psychology, including pioneering work by Sherif [25], Asch [26], and Milgram [27]. Recently, the notion of social influence has received considerable attention from a variety of fields including public health and political science [28, 29]. This work has demonstrated that behavior is not only influenced by those we interact with, but also by the people who interact with those we interact with (and so on), an idea we refer to as an influence cascade. The idea that social influence *cascades* through our social networks is a powerful idea that suggests potentially efficient and powerful ways to alter population-level behaviors [30].

Researchers have recently begun to investigate whether altruistic behavior can cascade through social networks. Fowler and Christakis recently reported a reanalysis [31] of a prior study [32] in which altruism was measured using the public goods game. In a public goods game, a group of players each receive an initial endowment [31]. Each player then chooses how much of this endowment to keep for herself and how much to contribute to the public good. The total contribution to the public good is multiplied (e.g., by 1.4) and then divided equally among the players. Contributing to the public good is considered altruistic because such contributions are personally costly but provide benefits to everyone in the group. Participants played six rounds of a public goods game in groups of four. During each round, participants were randomly assigned to new groups, with no two participants ever interacting more than once. Fowler and Christakis’ analyses revealed that participants’ contributions were affected by their partners’ contributions; participants who previously interacted with generous partners subsequently contributed more themselves. Critically, participants’ contributions were also influenced by participants they did not directly interact with; participants interacting with partners who previously interacted with generous partners subsequently contributed more themselves. Indeed, influence was found to extend up to three degrees: contributions were influenced by partners’ partners’ partners’ contributions. This indirect influence, an example of an influence cascade, was the critical finding of their study.

One unique strength of Fowler and Christakis’ results is that participants were randomly assigned to groups [31] (cf. [22, 33–35]) and were randomly regrouped after each round. This technique allowed the authors to avoid confusing factors such as homophily (people’s tendency to interact with those who are already similar) with true social influence. Nonetheless, the data used by Fowler and Christakis was observational in the sense that there was no experimental manipulation and thus could only provide an estimate of how different behavior (e.g., generous or selfish) would spread across social networks as a result of a causal intervention. In addition, the magnitude of the influence cascade was relatively small. For example, for each additional unit contributed by a partners’ partner, participants contributed only an additional 0.07 units.

Suri and Watts report a study that is superficially similar, in which participants again played a public goods game [36]. However, unlike the Fowler and Christakis study, Suri and Watts were primarily interested in the influence of network topology, a factor that has received much attention in the field (e.g., [7–9, 14–16]). In addition, Suri and Watts included an intervention in order to investigate the possibility of influence cascades. Their intervention consisted of a small number of “seed nodes”: participants under full control of the experimenters who always contributed maximal amounts in the public goods game. Suri and Watts found that their interventions resulted in direct influence; seed nodes did encourage their partners to contribute more. However, the interventions yielded no indirect influence; participants two degrees away from seed nodes (i.e., partners of the seed nodes’ partners) were no more generous than participants two degrees away from ordinary participants (i.e., partners of non-seed-node participants’ partners). Suri and Watts attribute the apparent discrepancy between their own findings and the findings of Fowler and Christakis [31] to methodological differences in the two studies. For example, in Fowler and Christakis, participants never interacted with the same partner more than once, whereas Suri and Watts’ participants played with the same partners repeatedly, for a total of 10 rounds. Suri and Watts detail how this leads to differing notions of network “distance” in the two studies. We would further note that the repeated interactions used by Suri and Watts make the evaluation of social influence somewhat difficult. That is, each player, including the seed nodes, had repeated interactions with multiple partners, which means that the number of paths through which influence could flow was quite large. These multiple paths leave open the possibility, among others, that any indirect influence could have been overwhelmed by the direct influences of other players. Furthermore, though Suri and Watts employed an intervention, unlike Fowler and Christakis [31], the seed nodes were exclusively generous; no selfish seed nodes were included in their design.

Gray and colleagues have recently reported a similar study in which participants instead played a dictator game [37]. In a dictator game, one player assumes the role of dictator and the other assumes the role of receiver. The dictator receives an endowment and decides how to split it between herself and the receiver. In Gray et al.’s study, participants played the role of the recipient on the first round and then played the role of the dictator on the second round, each round playing with a different player. When playing the role of the recipient, participants interacted with either a generous dictator (the dictator gave 100% of her endowment), an equitable dictator (the dictator gave 50% of her endowment), or a selfish dictator (the dictator gave 0% of her endowment). Results indicated that interacting with the selfish dictator influenced participants’ subsequent behavior. Specifically, participants that interacted with a selfish dictator subsequently contributed less than those interacting with generous or equitable dictators. Furthermore, subsequent behavior of those interacting with generous and equitable dictators did not differ. This finding suggests that selfish behavior, but not generous behavior, spreads via social interactions. We note that this finding may provide an additional explanation of the apparent discrepancies between the studies of Suri and Watts, who only investigated the influence of generous behavior [36], and Fower and Christakis, who included no intervention [31]. Gray et al. attribute their findings to affective mechanisms [37]. That is, participants who previously interacted with selfish individuals experienced more negative affect (e.g., anger), which subsequently led them to behave more selfishly. Unfortunately, Gray et al. do not provide evidence for this particular mechanism in experiments involving the dictator game, instead only measuring affect in experiments involving non-monetary exchanges (i.e., exchanges of labor). Furthermore, the nature of Gray et al.’s design precludes the possibility of evaluating indirect social influence.

Finally, a recent study by Tsvetkova and Macy [38] examined potential differences between being a direct recipient of others’ generosity and simply observing generous behavior. A subgroup of participants was first invited by experimenters to play what they referred to as the invitation game. Everyone invited to the game received a base payment and an additional bonus payment. Participants then had to decide whether they wanted to invite an additional, randomly chosen, participant to the game by donating the bonus payment. If a participant decided not to invite another participant, she kept both the base and bonus payments. If she invited another participant, she would keep only the base payment. Results indicated that being the recipient of altruistic behavior did encourage participants to generously forgo their bonus payment in order to invite further participants. However, merely observing the frequency of generous behavior within the larger population (but without directly receiving its benefits) actually discouraged participants from forgoing their bonus payment. Unfortunately, like Gray et al., the nature of Tsvetkova and Macy’s design precludes the possibility of evaluating cascades of altruism. Furthermore, as in Suri and Watts [36], only the contagion of generosity was examined; no intervention of selfish behavior was included.

### The Current Study

Prior research has suggested that altruistic behavior can exert direct social influence. However, there is conflicting evidence about indirect influence, creating uncertainty about whether altruistic behavior can cascade through social networks. The studies reviewed above each have notable methodological limitations. Further, findings are limited and unclear on whether selfish behavior spreads similarly to generous behavior. The current study attempted to investigate how far altruistic behavior can spread through social networks, whether selfish and generous behavior exert differentially powerful influences, and what cognitive mechanisms might underlie such influences.

**Fig 1:**
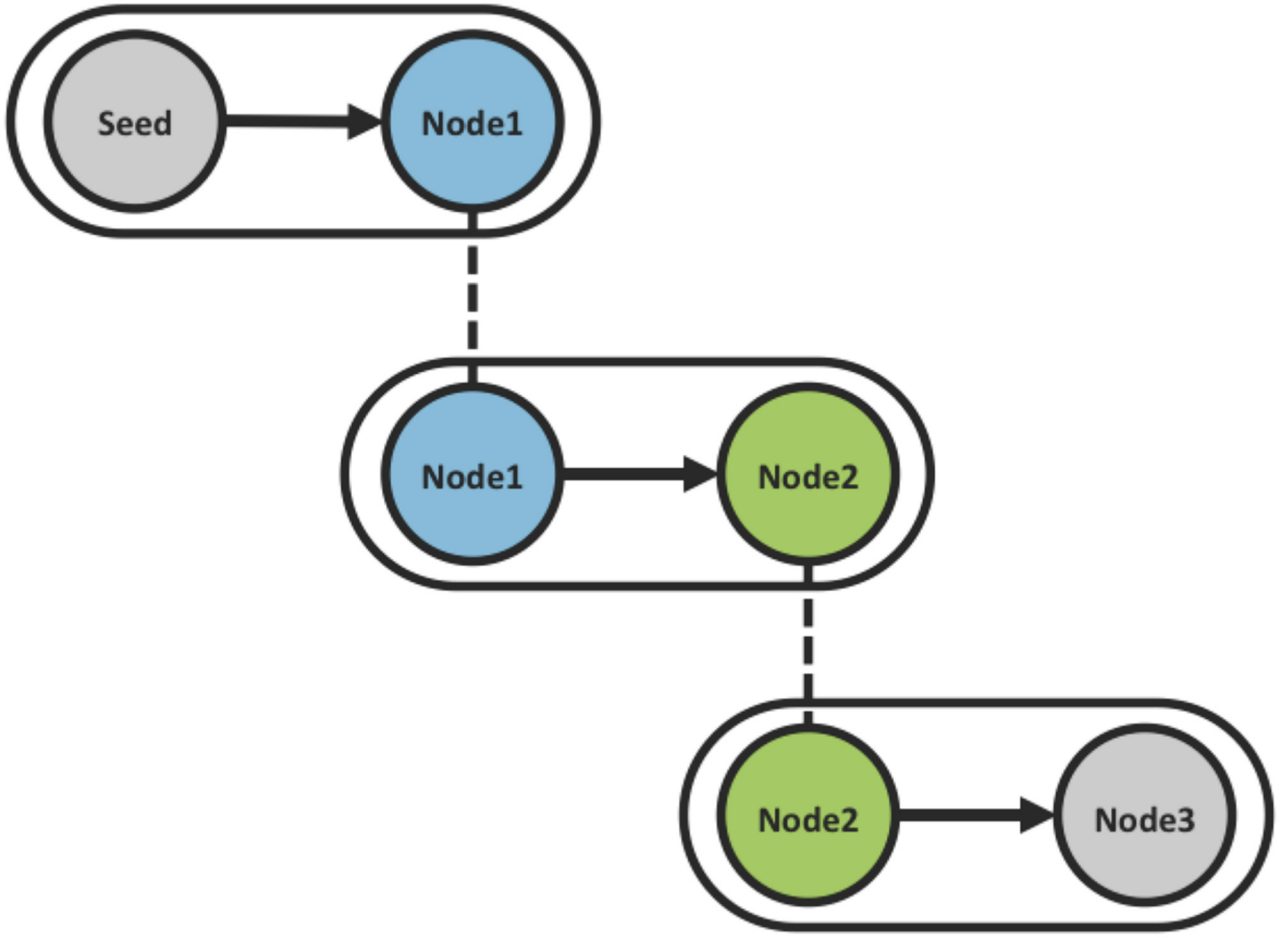
The network used in the current study. In each chain, the Seed Node first interacted with the Node-1 player. The Node-1 player then interacted with the Node-2 player. The Node-2 player then interacted with Node-3 player. Node-1 and Node-2 players were human participants, whereas the Seed Node and Node-3 players were under experimental control.

The networks used in the current study were chains, each with four nodes (see Fig 1). In these chains, the Seed Node first interacted with the Node-1 player in a single-round of the public goods game. The Node-1 player then interacted with the Node-2 player in a single-round public goods game. Finally, the Node-2 player interacted with the Node-3 player in a single-round public goods game. In this way, each Node-1 player and each Node-2 player played two rounds of the public goods game, interacting with a different partner in each round. Each of them made one choice before observing the behavior of a partner (i.e., in the first game) and made one choice after observing the behavior of a partner (i.e., in the second game).

This chain structure provides two main benefits. First, it allows for observation of both direct and indirect social influence. Second, it minimizes the possible paths of influence because each player can only be directly influenced by one other player and influence can only flow in one direction (e.g., Node-1 players only interact with Node-2 players *after* interacting with Seed Nodes, see [39] for use of similar structures).

To investigate the influence of both selfish and generous behavior, we manipulated the behavior of the Seed Nodes to be either selfish or generous. To investigate the nature and magnitude of both direct and indirect influences, we examined whether our manipulation of the Seed Nodes influenced the behavior of both Node-1 and Node-2 players. To investigate the possible mechanisms underlying any observed influence, we examined whether past interactions influenced participants’ expectations about subsequent partners and whether such expectations accounted for any social influences on behavior [40]. We also measured individual differences in social discounting (how people value rewards for others compared to rewards for oneself [41]) and delay discounting (how people value delayed rewards compared to immediate rewards [42, 43]) as potentially relevant factors that could shape altruistic behavior ([10, 11, 13, 44] provide evidence that social distance is related to altruistic behavior).

## Methods

### Ethics Statement

This study was approved by the Stony Brook University Institutional Review Board. Written informed consent was obtained from all participants.

### Participants

One hundred and twenty-five undergraduates from Stony Brook University participated in the experiment for partial course credit. Participants were randomly assigned to either the Node-1 or Node-2 position in either a generous or a selfish chain (note that Seed Node and Node-3 contributions were computer-generated). Overall, there were a total of 63 players in the generous condition (32 Node-1 players and 31 Node-2 players), and 62 players in the selfish condition (31 Node-1 players and 31 Node-2 players). In addition to course credit, participants also received a cash reward based on the points they earned in the public goods game. Earnings were ranged from 5 to 7 dollars.

### Tasks

#### Public goods game

The public goods game portion of the experiment consisted of instructions including an interactive demo, followed by a quiz (to ensure their understanding of the game), and culminated in two actual public goods games in which participants made choices that resulted in actual cash earnings. First, participants were told that they and another player would each receive 10 tokens at the beginning of each game, which can be invested into a public project. In each game, they and their partner could invest any amount of their 10 tokens into the project. The total investment was then multiplied by 1.5 and split equally between both players. A player’s final earnings per game were the sum of their portion of the earnings from the public project and the tokens that they did not invest. After the instructions, the participants interacted with a demo that allowed entry of two hypothetical contributions (one from the participant and another from a hypothetical player) and then displayed the step-by-step calculation of each player’s final earnings. Participants were told to play the demo game as many times as they wanted and to explore how different investments from each player would affect the final earnings. Next, the participants were given a quiz which consisted of four questions involving calculating final earnings following a pair of hypothetical investments (e.g., “In a hypothetical scenario, what are your final earnings if you invest 0 tokens and your partner invests 6 tokens?”) and three conceptual questions (e.g., “If the other person invests 10 tokens, how many tokens should you invest in order to maximize the other person’s final earnings?”) (see Appendix A for all the questions). Participants answered each question by circling one of four options. Participants correctly answering at least three computational questions and 2 conceptual questions were considered to understand the public goods game properly and to continue in the study. After the quiz, participants completed two public goods games. Before the games began, participants were told that they would be paid one dollar for every four tokens earned during the games. Final earnings were rounded up to the next nearest dollar.

#### Delay Discounting

This is a standard delay discounting task involving hypothetical rewards and is adapted from a previous measure [43]. The task measured participant’s preferences for different monetary rewards over time. On each trial, the participants were presented with a hypothetical choice between a smaller immediate monetary reward (e.g., “$40 now”) and a larger delayed monetary reward (e.g., “$55 in twenty-five days from now”). There were a total of 27 trials, wherein the response on each trial indicated a participants’ discounting rate for the delayed reward (e.g., in the aforementioned example, a preference for the delayed amount would indicate a discounting rate of less than or equal to 0.015). The measurable range of discounting rates for this task was from 0.0007 to 1.0 [42]. Discount rates were estimated by fitting an exponential discount model to individual participants’ data and maximizing the log-likelihood of the observed choices. Note that because the distribution of discounting rates is highly skewed, discounting rates used in all analyses were log-transformed.

#### Social Discounting

This is a standard social discounting task involving only hypothetical rewards and was adapted from a pen-and-paper task [45]. The task measured preferences for different monetary rewards delivered to people at varying social distances from oneself. Analogous to delay discounting, it reflects how people discount the value of rewards to others based on social distance. Participants were instructed to imagine a list of people arranged in order from their closest friend or relative at #1, to a distant acquaintance at #100. On each trial, the participants were asked to indicate a preference between two choices—an amount of money for themselves (e.g., “$40 to yourself”) or a larger amount for a person of a specific social distance (e.g., “$55 to person #25”). As in the delay discounting example, preference for the reward for another person in the above example indicates a discounting rate of exactly 0.015. The social discounting task also consisted of 27 trials. The rewards used in these trials were identical to those in the delay discounting task, and the social distance used were the same as the temporal delays in the delay discounting task (e.g., “person #25” rather than “25 days from now”).

### Procedure

Upon arriving, participants were sent to different individual testing rooms. This was done to minimize face-to-face interaction between participants and to allow for computer-generated partner behavior (e.g., Seed Node and Node-3 players) without the need for human confederates. Participants were told that they would play two games, each with a different partner. Nothing about partners’ previous behavior in the game was mentioned to participants. At no time did was the possibility that participants’ partners might be anything other than human mentioned. Participants were not given any information about the larger network (i.e., chain) in which they were embedded was provided.

Participants were given instructions individually and then completed a demo task. During the demo, participants had to click on corresponding boxes that represented their own and a hypothetical partner’s investments, entering integers from 0 to 10 using the keyboard. When they clicked on a “Finalize” icon, the computer would demonstrate the step-by-step calculation of each of their final earnings. When participants reported finishing the demo game, they were given the aforementioned quiz. After the quiz, participants were told that they would play two games, with a different partner in each game. They were also told that they would be paid based on their total earnings from the two games. For every four tokens they earned, they received one dollar.

The procedure of the actual games was identical to the demo except for two changes. First, to enhance the impression that participants were actually playing with other (human) participants, the computer showed each of the following messages for two seconds before each game started: “loading database…”, “connecting to server”, and “waiting for other player”. Second, after participants clicked “Finalize”, the computer revealed the investment from the partner and then the final earnings of each player. The computer did not show step-by-step calculation of the final earnings during the actual game. Third, in the second game, after participants finalized their contribution but before revealing the partner’s contribution, participants were asked how many tokens they expected their partner to invest.

During the first of the two public goods games, the partner’s contribution was always a pre-determined quantity. For Node-1 participants, the Seed Node contribution was pre-determined to be 9 tokens in the generous condition and 1 token in the selfish condition. For Node-2 participants, the partner contribution was pre-determined to be the actual contribution by the corresponding Node-1 participant in that chain. In the second game, the partner’s contribution was pre-determined to be 10 minus the contribution of participants’ partner in the first game. Having partners’ contributions be pre-determined not only allowed us to implement our experimental manipulation (i.e., contributions of the Seed Nodes), but also allowed us to have Node-2 players observe the contributions of their Node-1 partners without needing the constituent individuals to participate simultaneously (and alleviated needing human participants for the theoretically uninteresting Node-3 player).

After finishing the public goods game, participants completed the delay discounting task and the social discounting task in counterbalanced order. Participants were then paid and debriefed.

## Results

The layout of the results section is as follows. In our first set of analyses, we explore the potential existence of and mechanisms underlying the social transmission of altruistic behavior. These initial analyses essentially ignore the existence of our causal intervention and treat our dataset as though it were purely observational [31] and explore the possibility that all participants’ Game-2 contributions were influenced by their partners’ contributions in Game 1. In our second set of analyses, we directly evaluate the consequences of our causal intervention. Specifically, we explore whether Generous and Selfish Seed Nodes altered the Game-2 contributions of Node-1 players (i.e., direct influence) and Node-2 players (i.e., indirect influence).

### Does observed behavior influence subsequent contributions?

To examine whether Game-1 contributions influenced Game-2 contributions, we conducted regression analyses. Here and in subsequent regression analyses that use contributions or contribution-related measures as the dependent variable, we employ Tobit regression due to censoring (i.e., contributions could not be smaller than zero or larger than ten; see [31, 46] for prior use of such analyses in public goods experiments). As shown in Table 1a, both participants’ own Game-1 contributions and their partners’ Game-1 contributions were significant predictors of participants’ Game-2 contributions; social discounting was also a significant predictor (though the coefficient suggests a relatively minimal relationship), but delay discounting was not. These results suggest that participants’ contributions were influenced by the behavior observed in their previous partners. That is, the more their partners contributed in previous games, the more participants themselves contributed in later games. Furthermore, the significant predictive power of participants’ Game-1 contributions suggests that this influence led participants to revise, rather than discard, their own idiosyncratic tendencies.

**Table 1:** Results of the regression analyses.

(a)	Beta	p	(b)	Beta	p	(c)	Beta	p
Y = G2			Y = Exp			Y = G2		
G1	.75	< .001	G1	.28	< .01	G1	.64	<.001
Obs	.35	< .001	Obs	.36	< .001	Exp	.50	<.001
SD	-.07	.02	SD	.01	.67	SD	-.05	.08
DD	.10	.67	DD	.16	.45	DD	.13	.59

Y = criterion variable

G1 = participant’s game-1 contribution

G2 = participant’s game-2 contribution

Obs = observed contribution from partner in game 1

Exp = expectations about partners’ contributions

SD = social discount rate

DD = delay discount rate

We also explored whether the influence of behavior observed in Game 1 differed as a function of participants’ position in the chain. Specifically, we constructed a dummy variable representing whether participants were Node-1 players (and thus interacted with Seed Nodes in Game 1) or Node-2 players (and thus interacted with Node-1 players in Game 1). We then modified the above regression model to include the interaction between this dummy variable and their partners’ Game-1 contributions as an additional predictor. This interaction term was not significant, suggesting that the influence of observed behavior was equivalent across the two links within the chain.

### Does observed behavior influence subsequent expectations?

We next examined whether contributions observed in Game 1 influence participants’ expectations about their partners’ Game-2 contribution. As shown in Table 1b, both participants’ own Game-1 contributions and their previous partners’ Game-1 contributions were significant predictors of participants’ expectations about their partners’ Game-2 contributions; delay discounting and social discounting were not significant predictors. These results suggest that participants formed expectations about unfamiliar partners based on their own, prior contributions, but also on the contributions of their previous partners. The more generous their previous partners were, the more they expected new partners to contribute.

We next examined whether participants’ expectations about their partners’ contributions were related to their own contributions. As shown in Table 1c, both participants’ own Game-1 contributions and their expectations were significant predictors; delay discounting and social discounting were not significant predictors. These results suggest that participants’ Game-2 contributions were proportional to the behavior expected on the part of their new partner; the more participants’ expected their partners in game 2 to contribute, the more they themselves would contribute and vice versa. This finding is consistent with previous research [40].

### Do expectations mediate the relationship between observed behavior and subsequent contributions?

Given that participants’ expectations were predicted by past observations and predicted future contributions (and that past observations also predicted future contributions), it seems plausible that such expectations play a critical role in the social transmission of altruistic behavior. In order to further examine expectations as a putative psychological mechanism, we conducted a mediation analysis [47]. Specifically, we explored whether participant expectations mediated the relationship between partner contributions observed in Game-1 and Game-2 contributions. That is, we explore the extent to which the relationship between Game-1 observations and Game-2 contributions (reported in Table 1a) could be accounted for by participants’ expectations. To do this, we used the non-parametric bootstrap technique [48], suitable for use with Tobit models. A 1000-sample bootstrap (presented in Table 2) indicated that expectations did mediate the relationship between the previously observed behavior and participants’ subsequent contributions, and this mediator represented a substantial proportion of the total effect.

**Table 2:** Results of analyses investigating whether participants’ expectations regarding their partners’ contributions mediated the social influence effects.

Quantity	Mean	p
Mediation Effect	0.1133	< .001
Direct Effect	0.2045	.03
Total Effect	0.3178	< .001
Proportion Mediated	0.3566	< .001

### Did Seed Node contributions affect Node-1 participants?

So far, our analyses have largely ignored critical features of our experimental design, such as our causal intervention and the distinction between Node-1 and Node-2 players. In what follows, we focus on whether Seed Node behavior influenced the contributions of Node-1 players (direct influence) and Node-2 players (indirect influence). We evaluate each of these influences in two different, but complementary ways. The first approach we take is to simply evaluate the observed contributions made by players in our Generous chain with the observed contributions made by players in our Selfish chain. This analysis provides a very straightforward assessment of the magnitude of both direct and indirect influences generated by our experimental manipulation. The second approach we take is inspired by the analyses of Fowler and Christakis [31], in which participants’ behavior is predicted by regression models that included participants’ behavior in prior games. Such models control for a priori individual differences that might otherwise make influence difficult to detect. Though the analyses of Fowler and Christakis are the model’s most direct inspiration, similar analytic strategies have been adopted by others [22, 23]. Our own analyses consist of regression models constructed to disentangle the potential influence of the Seed Nodes from sources of variability that are unrelated to influence (e.g., pre-existing individual differences).

**Fig 2:**
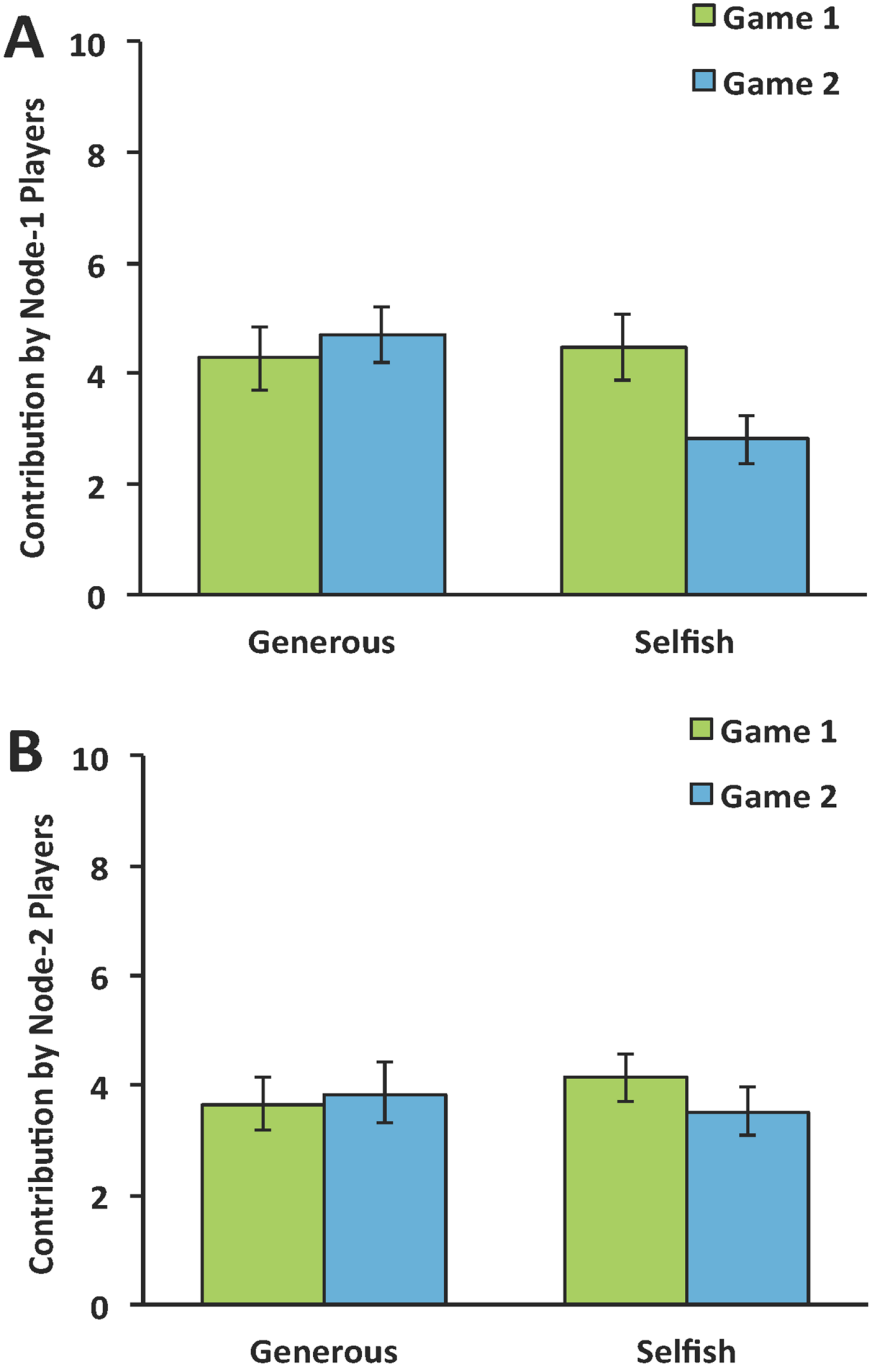
Contributions in Game 1 and Game 2 in Selfish and Generous conditions. (A) Contributions by Node-1 players. In Game 2, participants in the Selfish chain contributed significantly less than those in the Generous chain. Furthermore, participants in the Selfish chain contributed significantly less in Game 2 (after interacting with the selfish Seed Node) than in Game 1. (B) Contributions by Node-2 players. No effects were observed.

**Fig 3:**
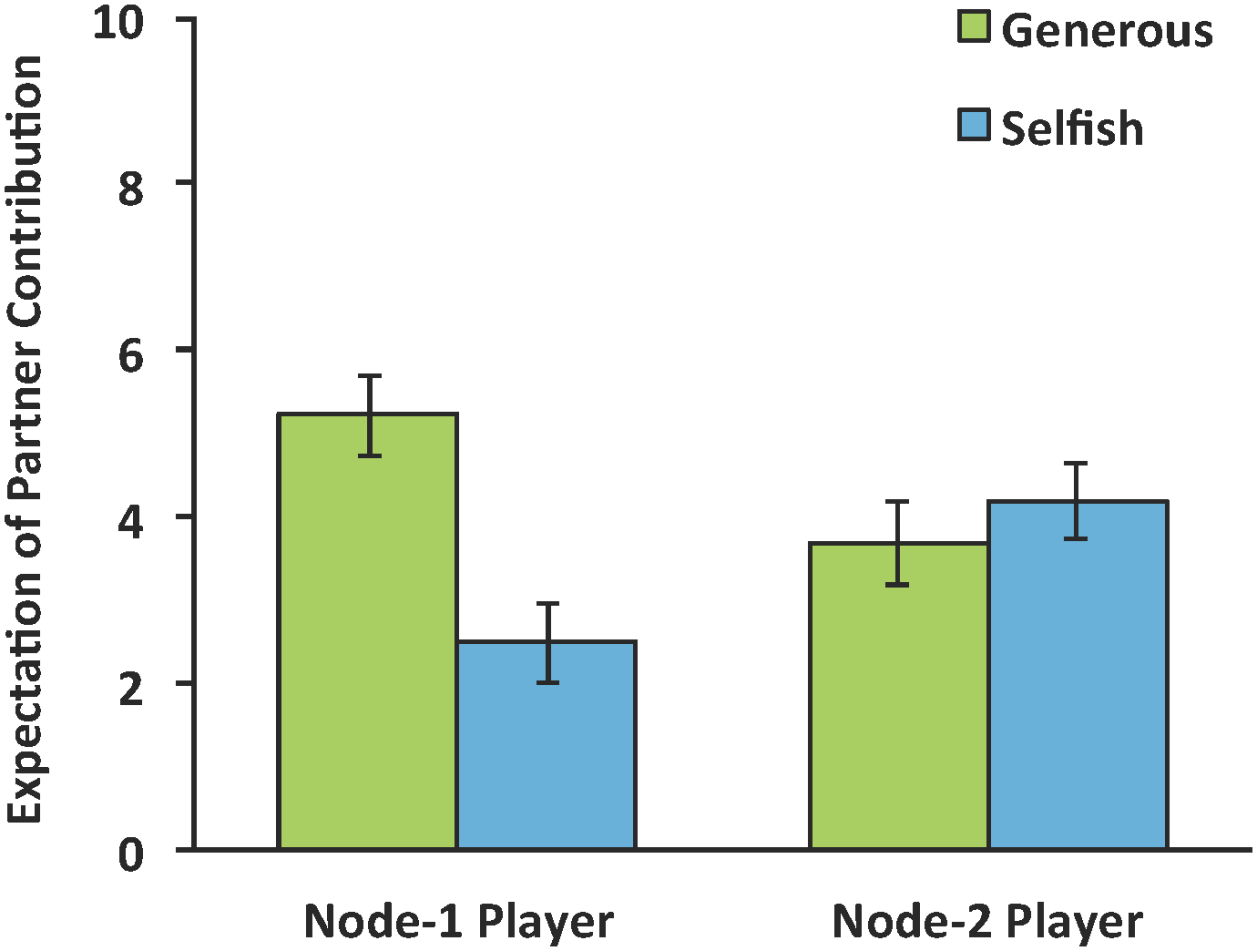
Participants’ expectations regarding partners’ contributions in Game 2. Node-1 players in the two chains held significantly different expectations. Node-2 players did not.

Contributions of Node-1 participants in the two games are plotted in Fig 2A. To examine whether the behavior of the Seed Nodes influenced Node-1 participants, we conducted a 2 (chain: Selfish vs. Generous) by 2 (game: Game 1 vs. Game 2) mixed ANOVA on Node-1 players’ contributions, with repeated measures on the latter factor. This analysis indicated no main effects, *p*’s > .15, but a significant interaction, *F*(1, 61) = 8.21, *p* < .01. In Game 2, participants in the Generous chain contributed more (M = 4.84, SD = 3.28) than participants in the Selfish chain (M = 2.90, SD = 2.83), *t*(61) = 2.51, *p* < .05 whereas no such difference was observed in Game-1 contributions, *t*(61) < 1, *p* > .7. These results confirm that differences between the Generous and Selfish chains were a consequence of our causal intervention (in the form of the Seed Nodes). Participants in the two chains were not different before observing Seed Node behavior, but diverged after observing the Seed Nodes’ contributions. In addition, participants in the Generous chains expected their partners to contribute more in Game 2 (M = 5.22, SD = 2.80) than participants in the Selfish chains (M = 2.48, SD = 2.67, *t*(61) = 3.96, *p* < .001, see Fig 3)

Given that each participant selected one contribution before observing the behavior of others (i.e., Game-1 contributions) and selected one contribution after observing the behavior of a partner (i.e., Game-2 contributions), we have the unique opportunity to directly quantify the influence of this observation at the level of individual participants. Analyses revealed that Node-1 participants in the Selfish chain contributed less in Game 2 (M = 2.81, SD = 0.02) than they did in Game 1 (M = 4.48, SD = 2.94, *t*(30) = 2.63, *p* < .05). This suggests that participants who previously interacted with a Selfish Seed Node were less willing to contribute when interacting with new partners. In contrast, Node-1 participants in the Generous chain contributed no more in Game 2 (M = 4.70, SD = 3.28) than they did in Game 1 (M = 4.28, SD = 2.45, *t*(31) = -1.25, *p* > .22). This suggests that participants who previously interacted with Generous Seed Nodes were no more willing to contribute when interacting with new partners. Taken together, these results suggest that observing selfish behavior has a greater impact on people’s behavior than observing generous behavior [37].

With the result of these analyses, we can estimate the magnitude of the direct influence Seed Nodes had on Node-1 participants. Node-1 participants contributed an additional 0.56 units after observing the contributions of Generous Seed Nodes and contributed 1.58 units less after observing the contributions of Selfish Seed Nodes. We can also compare the two chains to get an overall estimate. Seed Nodes in the Generous chains contributed 8 more units than Seed Nodes in the Selfish chains whereas Node-1 participants in the Generous chains contributed 1.89 units more than Node-1 participants in the Selfish chains in Game 2. Thus, for each additional unit the Seed Node contributed, Node-1 participants contributed an additional 0.24 units.

Our next analysis was also designed to evaluate whether Seed Nodes influenced the subsequent behavior of Node-1 participants, but to do so in a way that allows us to control for sources of variability that were unrelated to social influence (e.g., pre-existing individual differences). Thus, we regressed Node-1 participants’ Game-2 contributions on their own Game-1 contributions, their previous partners’ (i.e., the Seed Nodes’) Game-1 contributions, and participants’ delay and social discounting rates. Results (see Table 3) indicated that participants’ Game-2 contributions were related to both their own Game-1 contributions and their previous partners’ (i.e., the Seed Nodes’) Game-1 contributions. These results are consistent with those reported above, suggesting that participants that directly interacted with the Seed Nodes were influenced by the behavior they observed. Moreover, the resulting coefficients provide a direct estimate of the magnitude of this influence. Specifically, for each additional unit the Seed Node contributed, Node-1 participants contributed an additional 0.39 units. Though we will return to this point in the General Discussion, here we briefly note that this estimate is substantially higher than the estimate derived from the contribution averages.

**Table 3:** Results of the regression analysis predicting Node-1 participants’ contributions in Game 2.

	Beta	p
Y = Node-1 G2		
Seed	.39	< .001
Node-1 G1	.78	< .001
SD	-.15	.14
DD	.24	.42

Y = criterion variable

G1 = participant’s game-1 contribution

G2 = participant’s game-2 contribution

Seed = contribution by seed node

SD = social discount rate

DD = delay discount rate

### Did Seed Node contributions affect Node-2 participants?

Finally, we examined whether Seed Node contributions influenced Node-2 participants, which would suggest a cascade of selfish or generous behavior; whether our intervention exerted an indirect influence on Node-2 participants via Node-1 participants. Contributions by Node-2 Participants are plotted in Fig 2B. We conducted a 2 (chain: Selfish vs. Generous) by 2 (game: Game 1 vs. Game 2) mixed ANOVA on Node-2 participants’ contributions. The analysis revealed no significant main effects nor an interaction, *p*’s > .3. In Game 2, participants’ in the Selfish chain contributed just as much (M = 3.52, SD = 2.50) as those in the Generous chain (M = 3.81, SD = 3.34), *t*(30) < 1, *p* > .7. Indeed, to quantify the magnitude of influence we observed on Node-2 participants, we note that the difference between Generous Node-2 contributions and Selfish Node-2 contributions was only 0.29 despite an 8-unit difference between the Generous and Selfish Seed Nodes. In other words, for each additional unit the Seed Nodes contributed, Node-2 participants only contributed an additional 0.04 units. Furthermore, Node-2 participants’ contributions in Game 1 were indistinguishable from their contributions in Game 2 in both chains (*p*’s > .3). As for expectations, unlike Node-1 participants, Node-2 participants in the Generous chains (M = 3.68, SD = 2.77) and the Selfish chains (M = 4.19, SD = 2.56) expected their partners to contribute equivalent amounts in Game 2, *t*(61) = 3.96, *p* > .4).

**Fig 4:**
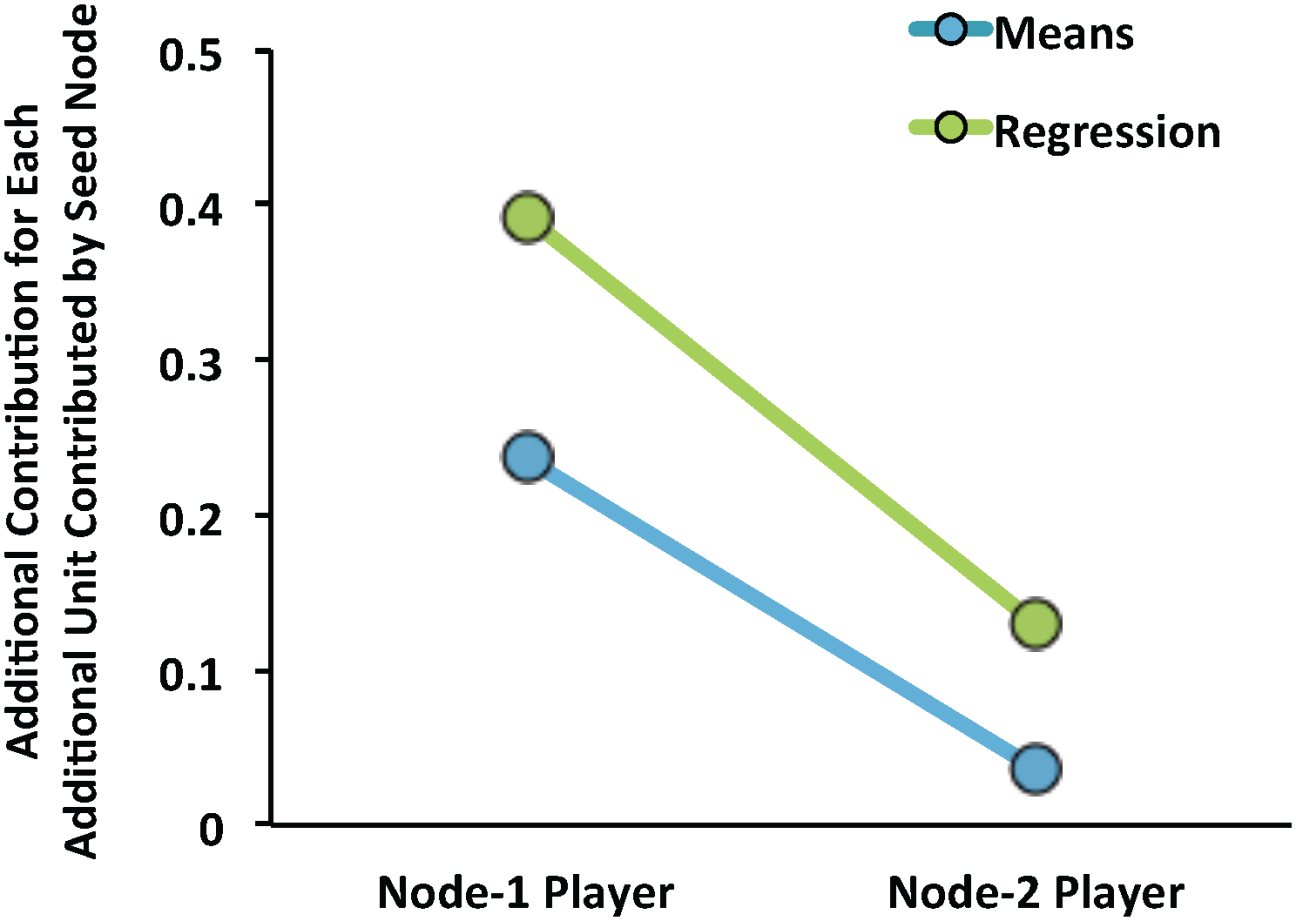
Influence on contributions for each additional unit contributed by Seed Nodes. The blue line shows estimates made using the mean contributions of participants in the Selfish and Generous chains. The green line shows estimates based on the regression analyses (as reported in Tables 3 and 4), which partial out individual differences in baseline contributions, delay and social discounting, etc.

Our next analysis was also designed to evaluate whether the Seed Node influenced the behavior of Node-2 participants, but to specifically compare such influence both before and after controlling for non-influence sources of variability. Specifically, we regressed Node-2 participants’ Game-2 contributions on Seed Node contributions (see Table 4). Results indicated that the Seed Nodes failed to exert any significant influence on the behavior of Node-2 participants. One potential explanation for this absence of indirect influence is that idiosyncratic factors such as participants’ pre-experimental preferences act as noise to eclipse the consequences of indirect social influence. For this reason, we evaluated two additional models, each of which included predictors representing such idiosyncratic factors. We first evaluated a model in which Node-2 participants’ preferences were controlled for (by including their game 1 contributions as well as their delay and social discounting rates). Finally, we investigated a model that added Node-1 participants’ game 1 contributions (importantly, these contributions were not observed by Node-2 participants). Each of these models revealed an increasing influence of the Seed Nodes’ contributions, though the influence was significant in neither model. We do note that this final model suggests that, for each additional unit the Seed Node contributed, Node-2 participants contributed an additional 0.13 units. In contrast to our initial analyses, results from these analyses suggest that controlling for an increasing number of potential sources of noise makes indirect influence increasingly apparent and that the magnitude of this indirect influence was ultimately estimated to be nearly four times larger than the estimate calculated above, using the average contributions (see Fig 4).

**Table 4:** Results of the regression analysis predicting Node-2 participants’ contributions in Game 2.

	Beta	p	Beta	p	Beta	p
Y = Node-2 G2						
Seed	.05	.72	.11	.36	.13	.27
Node-1 G1	--	--	--	--	.40	.02
Node-2 G1	--	--	.75	<.001	.79	<.001
SD	--	--	-.04	.18	-.03	.30
DD	--	--	-.15	.73	-.01	.98

Y = criterion variable

G1 = participant’s game-1 contribution

G2 = participant’s game-2 contribution

Seed = contribution by seed node

SD = social discount rate

DD = delay discount rate

## Discussion

The current study investigated both direct and indirect social influences on altruistic behavior. Our results indicate that altruistic behavior was directly influenced by those that participants directly interacted with. Furthermore, selfish behavior exerted greater influence than did generous behavior; participants became more selfish after interacting with selfish partners, but did not become more generous after interacting with generous partners. Moreover, the behavior of partners influenced expectations about subsequent partners such that participants expected others to behave as those they had previously observed. Our results suggest that this relationship may be a critical mechanism underlying the social transmission of selfish behavior.

One of the main questions of interest in the current experiment was whether social influences on altruistic behavior would lead to cascades of generosity/selfishness. That is, would participants be influenced by more distally related agents, such as partners’ previous partners? It is clear that participants at the end of our generous chains contributed no more than participants at the end of our selfish chains. Furthermore, participants in our generous chains contributed just as much before the opportunity to observe others’ behavior as they did after. These findings suggest that our experimental manipulation failed to generate influence cascades and that participants were in no way influenced by the behavior of participants further up the chain. In contrast, regression models controlling for participants’ pre-experimental individual differences seemed to indicate stronger indirect influence than models in which such differences were left uncontrolled. Taken together, our results suggest that a cascade was not evident, in part, because of variability in participants’ pre-existing preferences. To see why this is, we note that to generate systematic differences in the behavior of participants farther down the chain, our manipulation needed to produce generous/selfish behavior in absolute terms. Instead, the social influence we observed appeared to produce relative changes in altruistic behavior. That is, participants modified their prior behavior on the basis of the behavior observed in their partners (specifically, only when observing selfish behavior). For example, imagine a selfish Node-1 participant assigned to the Generous chain. She might contribute zero tokens in game 1 during which she observes that her partner (the Generous Seed Node) contributes nine tokens. If she is successfully influenced (in relative terms) she may contribute four tokens when playing with a Node 2 participant in game 2. If the Node-2 participant is generous and contributes ten tokens, she may conclude that the Node-1 participant is being selfish (in absolute terms) and thus contribute less in subsequent games. The fact that influence is operating in relative terms necessarily implies that the strength of our experimental manipulation is going to decay as it travels across a social network and may explain why we observed no indirect influence despite observing direct influence at each step our chains.

However, we were also able to estimate what the cascade would have looked like in the absence of individual differences by controlling for these idiosyncratic factors statistically (i.e., in the regression analyses). Indeed, once statistical noise was controlled for, analyses suggested that the difference between our chains should have been 1.7 (direct) to 3.6 (indirect) times larger than what we actually observed. For this reason, one should be wary of implications made about the effectiveness of interventions based on analyses of observational data sets [31]. This is particularly critical because the influence being quantified in such analyses is pertains to an implausible, “noise-free” environment.

Previous studies have found mixed evidence for the existence of indirect social influence. Two similar studies used the public goods game, with one finding indirect influences of up to three degrees (one’s partner’s partner’s partner) [31], and the other failing to find any indirect influence at all [36]. Like the study of Suri and Watts [36], the current results fail to find good evidence for indirect influence. In many ways, our study is much more straightforward than either of these two studies, but our results may also shed some light on the apparent discrepancy between these prior findings. Whereas these prior studies used rather complex networks, the network used in our study was designed to minimize the possible paths of influence; each participant only interacted with one partner before the potential influence from the partner was measured. Such a network was designed to maximize the chance of observing both direct and indirect influence and to do so without the need for statistical estimation. Furthermore, our design allowed us the unique opportunity to detect and quantify influence using both the more straightforward technique used by Suri and Watts [36], but also the more involved technique used by Fowler and Christakis [31]. Though Suri and Watts attribute the apparent discrepancies between the two studies to methodological differences, and network topologies specifically, we would note that the difference in analytic strategies appears to also be a potential factor. Indeed, when we simply compared the average contributions of our participants, as Suri and Watts did [36], our results suggest that participants were subject to direct but not indirect influences, just as reported by Suri and Watts. However, after partialling out participants’ individual differences, as Fowler and Christakis did [31], our estimates of indirect social influence were substantially larger.

Another potential concern is that there may be several different types of social influence. For example, in some circumstances influence may require the observation of a behavior from more than one source [49]. If social influences on altruistic behavior work in this manner, results from the current study (which employed simple chain structures) may not be easily extrapolated to more complex social structures. However, we note that prior behavioral studies [31, 36, 37] have all assumed that each individual is independently influenced by each of their peers. To the extent that this pervasive assumption holds, our results should apply directly to any social structure, regardless of complexity.

Most prior research investigating social influences on altruism has not distinguished between the specific nature of the observed behaviors [31, 36, 38] (see [37] for an exception). Our manipulation consisted of both selfish and generous behavior and our results indicated that interacting with a selfish individual has a greater influence. This finding is consistent with previous work that found that observing greedy behavior in a dictator game subsequently caused people to be greedier than observing equitable or generous behavior [37]. The fact that our results are consistent with previous findings [37] despite a variety of methodological differences suggests that the asymmetric influence of selfish and generous behavior may be robust (also see [50] for discussion on selfish and unselfish punishments). Fower and Christakis reported supplemental analyses to test for but failed to find evidence of such an asymmetry [31]. However, as discussed above, the lack of experimental control in Fowler and Christakis’ data places limits on the interpretation of such findings.

Gray et al. suggested that greed was more influential because it induced negative affect, which had a greater impact on behavior than the positive affect induced by generosity [37]. Though we cannot rule out emotion as a factor in our study, we note that our data provides evidence for an alternative expectation-based mechanism by which social interaction may influence altruistic behavior. Upon observing a partner exhibiting selfish behavior, our participants came to expect that future partners would also exhibit such behavior. In a public goods game, as in most social dilemmas, the most direct way to avoid exploitation by selfish agents is to behave selfishly yourself. In this way, the social influence of selfish behavior takes on a normative flavor (see [51] for discussion of the distinction between normative and non-normative cascades). Interestingly, the potential influence of generous behavior cannot easily be seen in the same normative terms. If you expect to interact with a generous individual, you are still better off being selfish. However, one can consider equity of various individuals’ payoffs (including one’s own), what economists refer to as Pareto efficiency. If you expect to interact with a generous individual, you can make the group better off (e.g., increase the average payoff) by being generous. Given that our generous intervention was largely unsuccessful, it appears that such considerations may be less automatic than the more defensive reaction to selfish behavior.

## Supporting Information

S1 Dataset (XLSX)
